# Advanced Electrocardiography Identifies Left Ventricular Systolic Dysfunction in Non-Ischemic Cardiomyopathy and Tracks Serial Change over Time

**DOI:** 10.3390/jcdd2020093

**Published:** 2015-05-13

**Authors:** Kerryanne Johnson, Stacey Neilson, Andrew To, Nezar Amir, Andrew Cave, Tony Scott, Martin Orr, Mia Parata, Victoria Day, Patrick Gladding

**Affiliations:** 1 North Shore hospital, Waitemata District Health Board, Takapuna, 0620 Auckland, New Zealand; E-Mails: Stacey.Neilson@waitematadhb.govt.nz (S.N.); Andrew.To@waitematadhb.govt.nz (A.T.); Nezar.Amir@waitematadhb.govt.nz (N.A.); Andrew.Cave@waitematadhb.govt.nz (A.C.); Tony.Scott@waitematadhb.govt.nz (T.S.); Martin.Orr@waitematadhb.govt.nz (M.O.); Mia.Parata@waitematadhb.govt.nz (M.P.); Victoria.Day@waitematadhb.govt.nz (V.D.); Patrick.Gladding@waitematadhb.govt.nz (P.G.); 2 Clinical Informatics Systems Group, North Shore Hospital, Waitemata District Health Board, Takapuna, 0620 Auckland, New Zealand; 3 Theranostics Laboratory, North Shore Hospital, Waitemata District Health Board, Takapuna, 0620 Auckland, New Zealand; 4 Auckland Bioengineering Institute, University of Auckland, 0620 Auckland, New Zealand

**Keywords:** left ventricular systolic dysfunction, advanced ECG, non-ischemic cardiomyopathy, echocardiography

## Abstract

Electrocardiogram (ECG)-based detection of left ventricular systolic dysfunction (LVSD) has poor specificity and positive predictive value, even when including major ECG abnormalities, such as left bundle branch block (LBBB) within the criteria for diagnosis. Although machine-read ECG algorithms do not provide information on LVSD, advanced ECG (A-ECG), using multiparameter scores, has superior diagnostic utility to strictly conventional ECG for identifying various cardiac pathologies, including LVSD. Methods: We evaluated the diagnostic utility of A-ECG in a case-control study of 40 patients with LVSD (LV ejection fraction < 50% by echocardiography), due to non-ischemic cardiomyopathy (NICM), and 39 other patients without LVSD. Diagnostic sensitivity and specificity for LVSD were determined after applying a previously validated probabilistic A-ECG score for LVSD to stored standard (10 s) clinical 12L ECGs. In 25 of the NICM patients who had serial ECGs and echocardiograms, changes in the A-ECG score *versus* in echocardiographic LV ejection fraction were also studied to determine the level of agreement between the two tests. Results: Analyses by A-ECG had a sensitivity of 95% for LVSD (93% if excluding *N* = 11 patients with LBBB) and specificity of 95%. In the 29 NICM patients without LBBB who had serial ECGs, sensitivity improved to 97% when all ECGs were considered. By comparison, human readers in a busy clinical environment had a sensitivity of 90% and specificity of 63%. A-ECG score trajectories demonstrated improvement, deterioration or no change in LVSD, which agreed with echocardiography, in 76% of cases (*n* = 25). Conclusion: A-ECG scoring detects LVSD due to NICM with high sensitivity and specificity. Serial A-ECG score trajectories also represent a method for inexpensively demonstrating changes in LVSD. A-ECG scoring may be of particular value in areas where echocardiography is unavailable, or as a gatekeeper for echocardiography.

## 1. Introduction

Since the advent of the electrocardiogram (ECG) over a century ago, its utility as a cost-effective and accessible diagnostic tool has been proven repeatedly. Through the application of rules and recognition of certain patterns, expert readers of ECGs can infer a wide variety of cardiac diagnoses. For these reasons the ECG has become a standard of care in the investigation of patients with suspected left ventricular systolic dysfunction (LVSD), and is a particularly valuable tool in primary healthcare [1].

Due to the abundance of patients presenting with dyspnoea, but relative inaccessibility of echocardiography and delays in laboratory testing, the ECG represents one of the most cost-effective point-of-care diagnostic instruments available in the community. Its utility however is limited by the experience of its users and quality of machine-read algorithms, which are often dismissed due to their perceived poor diagnostic quality. However modern advances in digital recording, signal processing, and machine learning have significantly improved accuracy of diagnostic ECG algorithms.

Advanced ECG (A-ECG) analysis utilises a number of these processes, but also typically derives from standard 12L ECG recordings results from additional techniques such as “3-dimensional” (spatial/spatiotemporal) ECG [2,3,4,5]; detailed studies of waveform complexity by singular value decomposition (SVD) [4,6,7,8] and, when longer recordings are available, also high-frequency QRS ECG [9]; and beat-to-beat QT variability (QTV) [10,11,12,13] and R-wave to R-wave variability (RRV) [14,15]. By combining the most applicable results from these advanced techniques into scores that rely on large underlying ECG databases and advanced statistics [16], diagnostic yield is increased substantially [17]. In addition as these databases grow, with additional cases and clinical feedback, iterative learning is facilitated and a virtuous circle completed.

In this retrospective case-controlled study, we applied a previously validated A-ECG score for LVSD [17] to the stored digital 12L ECG recordings of patients with a clinical diagnosis of non-ischemic cardiomyopathy (NICM), and to corresponding recordings from randomly selected matched patients who had normal echocardiograms. We used the electronically stored echocardiographic and other clinical information to evaluate the diagnostic accuracy of A-ECG for NICM.

## 2. Methods

### 2.1. Patient Selection

A local review of the study protocol by the North Shore Knowledge Centre (Auckland, New Zealand) classified this report as an observational review of electronic clinical records and therefore exempt from Ethics review (Ethical Guidelines for Observational Studies 2012. Health and Disability Ethics Committees of New Zealand) [18].

Patients who had undergone an echocardiogram with a referral stating “non-ischemic cardiomyopathy” or “NICM” were identified using a SQL server search of an Excelera (Philips Healthcare, Best, The Netherlands) database. Their data were included in the study if ≥2 serial echocardiograms had been performed. Controls were identified by choosing other patients from within the same database who had had clinical echocardiograms read as “normal”. Corresponding digital 12-lead ECGs were taken from Epiphany’s Cardio Server ECG management software (Version 3.2.3.1, Epiphany Healthcare, Midlothian, VA, USA), de-identified, and converted to an analysable, binary format by using an Octave script, then sent to Advanced ECG Services (Nicollier-Schlegel SARL, Trélex, Switzerland, https://aecg.ch) for A-ECG analyses.

### 2.2. A-ECG Analysis

Because the de-identified 12-lead ECGs were standard clinical ECGs containing only 10-s worth of information, A-ECG analyses were limited to only those parameters that can be accurately assessed from 10-s recordings. Analyzed parameters included those derived from signal averaging of all QRS and T complexes within the 10-s recordings by using software originally assembled at NASA [8,17] to generate results for: (1) several spatial (derived 3-dimensional) ECG parameters, including the spatial mean and peaks QRS-T angles, the spatial ventricular gradient, and various spatial waveform azimuths, elevations, and time-voltages [17], all derived by using the Frank-lead reconstruction technique of Kors *et al*. [19]; (2) parameters of QRS and T-waveform complexity derived by SVD that can be reproducibly obtained from 10-s ECGs, for example the principal component analysis (PCA) ratio ([8] and the dipolar voltage equivalents [4,17]) of the QRS and T waveforms; and (3) the most applicable parameters from the conventional scalar 12-lead ECG. The majority of these parameters and their related detailed methods have been described in previous publications [8,17,19,20]. To specifically judge the presence *versus* absence of LVSD by A-ECG, and then to also qualitatively follow the status of LVSD by A-ECG over time, we utilized a previously validated A-ECG score for LVSD that was originally constructed on the basis of multivariate logistic regression of A-ECG data from a larger data set of previous patients with known LVSD [17].

Results for this 5-component score are also derivable from the standard ~10-s 12-lead ECG recordings utilized in the present study. The score specifically incorporates results from a patient’s spatial mean QRS-T angle, derived Frank Z-lead integral, total (12-lead) QRS voltage, and SVD-derived QRS-wave nondipolar and T-wave dipolar voltages [17]. The exact coefficients utilized in the score can also be found within Supplemental Table 2 of [17].

### 2.3. Human Reader Sub-Study

A sub-study was also conducted wherein four human readers were also asked to provide a diagnosis for a random sample of 22 conventional 12L ECGs taken from all the ECGs in this study. 10 of these ECGs were from patients with NICM, and 12 were from control patients without NICM. Two respondents were cardiologists and two were general practitioners. The number of ECGs was reduced to limit the impact of time on clinicians with other clinical duties. For all 22 ECGs, the respondents were posed a single hypothetical patient with nonspecific breathlessness and instructed to provide a diagnosis of normal *versus* abnormal ECG. Moreover, for any ECG considered abnormal, they were asked to further segregate it into a most likely diagnostic category from amongst the following four choices: (a) coronary artery disease; (b) left ventricular hypertrophy or hypertrophic cardiomyopathy; (c) ischemic or non-ischemic cardiomyopathy (*i.e.*, LV ejection fraction < 50%); or (d) Long QT syndrome (LQTS) or other channelopathy.

### 2.4. Echocardiography

Echocardiograms were performed as part of clinical care on a GE Vivid 7 ultrasound machine. All echocardiograms were performed by an experienced trainee sonographer under cardiologist (ASE level III) supervision. The dates and data for each study were entered into a database and compared over serial assessment and with serial ECGs. Simpson biplanes estimates of ejection fraction were performed on all studies.

### 2.5. Statistics

Diagnostic sensitivity and specificity for LVSD (LV ejection fraction <50%) were determined after applying the aforementioned validated A-ECG score for LVSD [17] and the human reader results to the analyzed 12L ECGs. Cochran’s Q test was used for determining differences in accuracy between cardiologists, general practitioners and automated A-ECG in correctly identifying patients with *versus* without LVSD. For patients who had serial echocardiograms (LV ejection fractions) and corresponding serial A-ECG scores, the respective serial results were also characterized as improved, worsened or unchanged (i.e., when directional change was less than ±10%), thus allowing for qualitative comparison between directional changes in LV ejection fraction and the A-ECG score. Because the A-ECG score for LVSD was originally designed to determine, in a simple binary fashion, whether or not LVSD (defined as LV ejection fraction < 50%) was present, and not to attempt to track LV ejection fraction quantitatively [17], we performed a quantitative correlation between the two continuous parameters for academic purposes only. Finally, we also obtained A-ECG-related linear discriminant analysis (LDA) plots for purely descriptive purposes.

## 3. Results

### 3.1. Cases

Forty patients with LVSD due to clinically specified non-ischemic cardiomyopathy (NICM) were identified through the database search along with 39 controls without LVSD. Baseline patient characteristics are shown in Table 1 and Table 2.

**Table 1:** Patient characteristics.

	Cases (*n* = 41)	Controls (*n* = 38)	*p*-Value
Age (mean/SD)	57 (14)	44(19)	0.76
Type 2 DM (%)	9 (22)	2 (5)	0.06
HTN (%)	18 (44)	9 (24)	0.1
Current Smoker (%)	7 (2)	6 (16)	0.07
Ex-smoker (%)	15 (37)	9 (24)	0.3
IHD (%)	2 (5)	2 (5)	0.6
Dyslipidaemia (%)	12 (29)	7 (18)	0.37
PVD (%)	1 (2)	-	0.81
CVA/TIA (%)	4 (10)	-	0.14
AF (%)	12 (29)	1 (3)	0.005
Alcohol excess (%)	3(7)	-	0.29
Mental Health Dx (%)	3 (7)	3 (8)	0.8
Substance abuse (%)	2 (5)	-	0.49
Gout (%)	6 (15)	3 (8)	0.54
CKD (%)	2 (5)	2 (5)	0.6
Obesity (%)	21 (51)	11 (29)	0.08

DM: Diabetes, IHD: Ischemic heart disease, PVD: Peripheral vascular disease, CVA/TIA: Cerebrovascular accident/Transient ischemic attack, AF: Atrial fibrillation, Alcohol excess ≥ 21 standard drinks/week in a male or >14 in a female. CKD: Chronic kidney disease, Obesity = Body Mass Index > 30.

Nineteen patients underwent cardiac MRI for investigation of their cardiomyopathy. NICM was confirmed in 18 of these patients with only one patient also having myocardial scar consistent with the presence of coronary artery disease.

Twenty six patients underwent a coronary angiogram. Six of these were found to have moderate (≤50%–69%) to severe coronary artery disease (≥70%), eight had no disease and the remaining 12 had trivial to mild disease. Three of the six patients with moderate to severe coronary disease had undergone a cardiac MRI showing NICM, and the remaining three had a clinical diagnosis of NICM. Only one patient within the cohort was ultimately considered to have a mixed non-ischemic and ischemic cardiomyopathy based on information that became available, only after study completion.

**Table 2:** Patient medication and left ventricular (LV) ejection fraction at each echocardiogram date.

Ejection Fraction (mean/SD)	Echo 1 (*n* = 41)		Echo 2 (*n* = 41)		Echo 3 (*n* = 21)		Controls (*n* = 38)	
	25% (9)		31% * (11)		38% ^¥^ (13)		55%–60%	
	Drug Rx	Max dose	Drug Rx	Max dose	Drug Rx	Max dose	Drug Rx	Max dose
Betablocker (%)	15 (37)	2 (5)	39 (95)	14 (34)	17 (81)	12 (57)	8 (21)	1 (3)
ACEI (%)	17 (41)	7 (7)	30 (73)	12 (29)	13 (62)	7 (33)	6 (16)	2 (5)
ARB (%)	4 (10)	2 (5)	10 (24)	2 (5)	5 (24)	3 (14)	2 (5)	2 (5)
CCHB (%)	5 (12)	1 (2)	2 (5)	-	3 (14)	-	6 (16)	2 (5)
Spironolactone (%)	9 (22)	7 (7)	24 (59)	19 (46)	11 (52)	7 (33)	-	-
Digoxin (%)	2 (5)	-	4 (10)	-	4 (19)	-	-	-
Loop Diuretic (%)	9 (22)	-	27 (66)	-	11 (52)	-	-	-
Thiazide diuretic (%)	2 (5)	2 (5)	1 (2)	1 (2)	-	-	1 (3)	1 (3)
Statin (%)	10 (24)	2 (5)	18 (44)	1 (2)	6 (29)	1 (5)	9 (24)	1 (3)
Aspirin (%)	13 (32)	12 (29)	12 (29)	12 (29)	1 (5)	1 (5)	7 (18)	7 (18)
Warfarin (%)	3 (7)	-	12 (29)	-	7 (33)	-	-	-
Dabigatran (%)	-	-	2 (5)	-	1 (5)	-	-	-
Dipyridamole (%)	1 (2)	1 (2)	1 (2)	1 (2)	-	-	-	-
Amiodarone (%)	1 (2)	-	3 (7)	-	-	-	-	-
Clopidogrel (%)	-	-	1 (2)	-	-	-	1 (3)	-
ISMN (%)	-	-	-	-	-	-	1 (3)	-

*t* test comparison between Echo1 and Echo 2, * *p* = 0.004; and Echo 2 and Echo 3. ^¥^ *p* = 0.01.

The baseline characteristics for the control (non-NICM) group, including administered medications, are also shown in Table 1 and Table 2. The median number of days between the ECG and echocardiogram in controls was 81 days (range 1 to 758).

The maximum or target dose has been defined as follows: BB: Carvedilol 25 mg bd, Metoprolol 190 mg. Bisoprolol 10 mg. ACEI: Accupril 20mg bd, Lisonopril 20 mg Enalopril 20 mg. ARB: Losartan 50 mg, Candesartan 32 mg. Diuretics: Spironolactone 25 mg, Bendrofluazide 2.5 mg, Frusemide NA. CCHB: Diltiazem 360 mg, Amlodipine 10 mg, Felodipine 10 mg. Anti-arrhythmics: Digoxin NA, Amiodarone NA. Statins: Simvastatin 80 mg, Atorvastatin 80 mg. Anticoagulant/platelets: Aspirin 100 mg, Dipyridamole 25 mg bd, Clopidogrel 75 mg, Warfarin NA. Where the thiazide diuretic is in the form of a combination with ACEI it is assumed to be at maximum/target dose

The patients’ ECGs were obtained both before and after echocardiography. The median number of days between the first echocardiogram and ECG was 6 (range 0 to 114). The median number of days between the first and second echocardiogram was 321 (range 58 to 1287). The median number of days between the second echocardiogram and second ECG was 14 (range 0 to 147).

### 3.2. A-ECG

**Figure 1:**
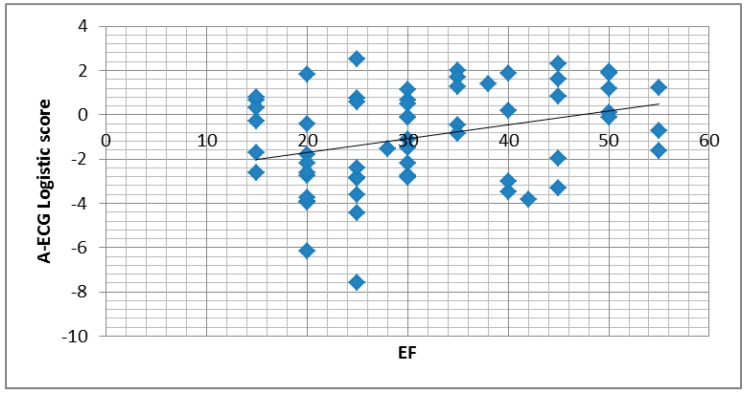

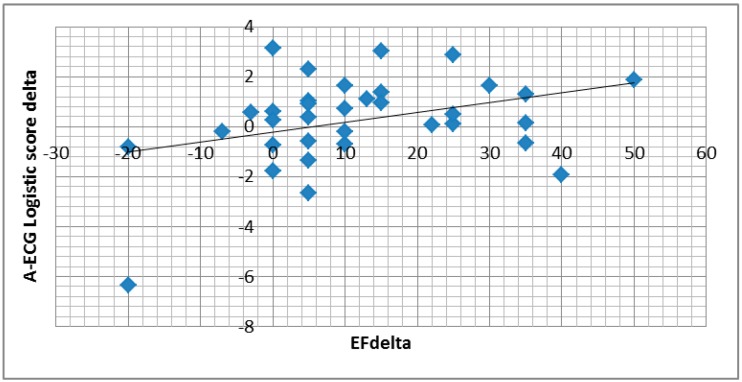
(Top): Baseline ejection fraction (EF) *versus* baseline A-ECG logistic score for left ventricular systolic dysfunction (LVSD), in all serial NICM patient ECGs; and (Bottom) Change in EF *versus* change in A-ECG logistic score.

Eleven of the patients with NICM had LBBB such that their ECGs could not be fully analysed for A-ECG. In the remaining patients (*N* = 68 total: 29 patients with NICM and 39 controls), A-ECG had a sensitivity of 93% for LVSD (95% if including the *N* = 11 patients with LBBB) and specificity of 95%. In the 29 NICM patients without LBBB who had serial ECGs, sensitivity of A-ECG also improved to 97% when all serial ECGs were considered. Trajectories in the A-ECG score for LVSD demonstrated improvement, deterioration or no change in LVSD, which agreed with changes in echocardiographic LV ejection fraction, in 76% of cases (*n* = 25). The quantitative correlation between the results of the A-ECG score for LVSD and the echocardiographic LVEF was r = 0.32 (*p* = 0.01). The correlation between the change in the A-ECG score for LVSD and the change in the LV ejection fraction between the first and second echocardiograms and A-ECGs (i.e., in patients who had serial results for both) was r = 0.35 (*p* = 0.01) (Figure 1). Fourteen patients had atrial fibrillation, a condition that did not preclude A-ECG scoring unless an LBBB was concomitantly present.

### 3.3. Human Reader Sub-Study

The human readers’ sensitivity and specificity for correctly designating, on the subset of 22 conventional 12L ECG tracings, the presence *versus* absence of LVSD through calls of “abnormal ECG”, and also for correctly designating the appropriate (*i.e.*, ultimately clinically proven) diagnostic category, are listed in Table 3. For both types of diagnosis, the performance of the cardiologists tended to be better than that of the general practitioners, although this difference was not statistically significant (Cochran’s Q = 1, df = 3, *p* = 0.8). However, the performance of the A-ECG score for LVSD was statistically significantly better than that of the clinical readers (Cochran’s Q 12.8, df = 4, *p* = 0.01).

**Table 3:** Diagnostic accuracy of cardiologists and general practitioners assessing for LVSD.

Multiple Diagnoses	Cardiologists	General Practitioners
Sensitivity	25%	15%
Specificity	71%	42%
**Binary Diagnosis (Normal/Abnormal)**	**Cardiologists**	**General Practitioners**
Sensitivity	90%	85%
Specificity	63%	58%

Average sensitivity and specificity for two readers reporting on a random sample of 22 ECGs from the overall cohort.

### 3.4. Controls

**Figure 2:**
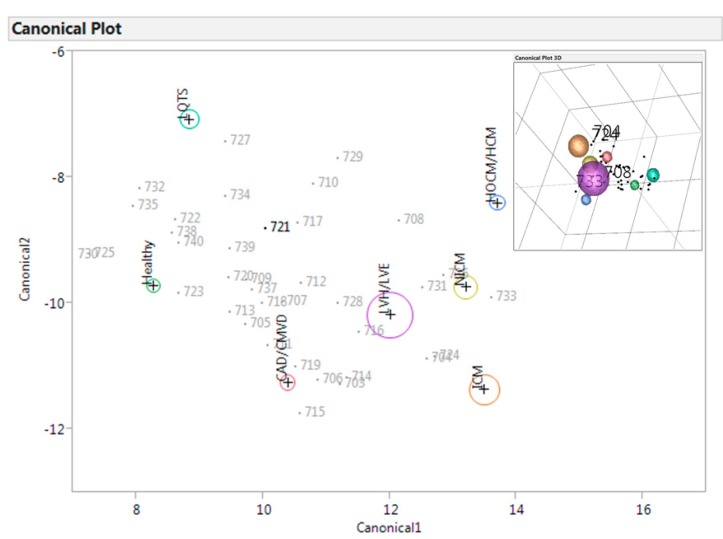
2D and 3D Linear Discriminant Analysis plot of control ECGs. Each individual control (non-NICM) patient’s A-ECG result as denoted by a number plotted within a 2-dimensional (2D) linear discriminant space. Probability of the presence of a given disease state is grossly demonstrated by the relative proximity of the patient's given number to a given disease state (larger circles), the latter being derived from a pre-existing database of A-ECG information from thousands of earlier patients with known, imaging-proven cardiac health or disease. Note however that because this is a 2D representation of a 3D space (right upper inset), a patient’s number may sometimes have the appearance of proximity to a disease circle in the x and y planes shown, but in the z plane the distance may actually be closer to some other sphere. CAD/CMVD = Coronary Artery Disease and/or Coronary Microvascular Disease Population; LVH/LVE = Left Ventricular Hypertrophy or Enlargement population; HOCM/HCM = Hypertrophic Cardiomyopathy population; NICM = Non-Ischemic Cardiomyopathy population; ICM = Ischemic Cardiomyopathy population; and LQTS = Long QT Syndrome (LQTS) population, respectively.

Of the 39 controls, 18 had abnormalities reported by A-ECG that were not related to LVSD. Two of these had interventricular conduction delays (QRS > 120 ms) such that their ECGs could not be fully analysed for A-ECG. A linear discriminant analysis (LDA) depicting the control population’s A-ECG results in 2D and 3D space is shown in Figure 2. In the LDA, the probability of the presence of a given disease state is grossly demonstrated by the relative proximity of the patient's given study number to a given disease state (larger circles), the latter being derived from a pre-existing database of A-ECG information from thousands of earlier patients with known, imaging-proven cardiac health or disease. Seven of the patients in the control group had A-ECG-related LDA results reporting a high probability of coronary artery disease. Of these, 6 patients had one or more cardiovascular risk factors. The 7th patient had late gadolinium enhancement on cardiac MRI and a diagnosis of myocarditis. The most abnormal A-ECG from this subgroup suggested a previous inferior myocardial infarction, which was confirmed by observation of regional wall motion abnormalities on echocardiography and by prior history.

The A-ECG LDA suggested a high probability of LV hypertrophy or enlargement in 5 other controls without NICM. One of these controls had a previous repair of a ventricular septal defect but no evidence of LV hypertrophy on echocardiography. Another had suspected Marfan’s syndrome with slender body habitus and a history of ablation of a Wolf-Parkinson-White pre-excitation pathway. The remaining three patients had hypertension.

Two of the controls without NICM had A-ECG patterns consistent with the Long QT syndrome, despite having QTc intervals within the normal range. Both of these patients had a history of recurrent syncope. One of these patients was non contactable while the other was recalled to clinic for possible genetic and other testing. Of all the patients in this cohort, 7 (18%) had conditions wherein genetic information might have aided diagnosis.

## 4. Discussion

In this retrospective case-control study, we prospectively further validated, in a group of patients with *versus* without NICM, the diagnostic utility of a previously published A-ECG score for LVSD [17]. In our patients, the A-ECG score had a sensitivity of 93% for LVSD (95% if including *N* = 11 patients with LBBB) and specificity of 95%. Thus screening by A-ECG would have accurately identified nearly all patients with existing LVSD due to NICM, distinguishing them from patients with normal LV ejection fraction and allowing the identification by ECG of those who needed more extensive cardiac investigations. Secondarily, we also demonstrated the same A-ECG’s scores qualitative ability to serially track changes in LVEF over time, based on the 76% correspondence between its results, when classified as improved, worsened or unchanged, with corresponding results from serial echocardiography.

Although the value of the ECG in the diagnosis of structural heart disease, such as ischemic heart disease, is well established, its utility in diagnosing LVSD is less so. The ability of the ECG to predict the presence of LVSD has been evaluated in a number of studies involving ECGs read both by humans and machine [1,21,22,23]. However with both types of readings the prediction of LVSD was ultimately allowed by indirect means, e.g., the presence of atrial fibrillation, or previous myocardial infarction or major nonspecific ECG abnormalities such as axis deviation or left bundle branch block (LBBB). Hence although the presence of a human-read major ECG abnormality can have high sensitivity for LVSD (90%–94%), the specificity is poor (58%–61%) [1,23]. In relation to our own sub-study of human-read ECGs, it is worth noting that most previous studies have asked readers to sort ECGs into normal or abnormal categories, using abnormality as a surrogate for LVSD. So although the sensitivity for the presence of LVSD appears high for human readers, this only applies to the detection of ECG abnormalities in the breathless patient, and hence *prediction* of LVSD presence, rather than its diagnosis. In this case-control study, we further validated not only A-ECG scoring’s equivalent sensitivity for LVSD (NICM) compared to human reading of conventional ECGs, but also its superior specificity. Furthermore A-ECG results as applied to discriminant analyses provided a spectrum of further diagnoses for each individual ECG, which could not be matched in sensitivity or specificity by human readers who did not have further clinical information.

Two factors are noteworthy in this respect. First, the sensitivity of a human read ECG is highly influenced by experience, with reported sensitivity of 87% for less experienced readers [1]. By comparison A-ECG provides consistency unmatched by a human reader, who may also have external pressures of time, workload and fatigue. Second, LBBB is included as a major ECG abnormality in many of the human read ECG pathways to triage echocardiography [23]. By including patients with LBBB in the present cohort, the sensitivity of A-ECG in the diagnosis of LVSD would increase to 95%. And even when excluding patients with LBBB, sensitivity would also further increase to 97% if/when all serial ECGs were scored by A-ECG.

Whilst many ECG parameters, such as the QRS duration [24], are known to alter with left ventricular dilation and dysfunction, modest changes in these continuous parameters are not easily perceptible to human readers. Computerised ECG scores have been described since the 1970s and 1980s and initially showed, in small populations of patients with ischemic cardiomyopathy, remarkable sensitivity (93%) and specificity (88%) for LVSD as well as strong correlations with LVEF (r = 0.8 to 0.9) [21,22]. However, the performance of these older, strictly conventional ECG scores was notably diminished during subsequent attempts at validation with further (larger *N*) cases of ischemic cardiomyopathy [25], such that they were never translated into clinical practice [26]. In addition, these scores focused only on detecting LVSD in the context of ischemic cardiomyopathy, emphasising changes in Q and R wave amplitudes to estimate the extent of infarction and thereby an ejection fraction. As such, they also never demonstrated any effectiveness for detecting nonischemic cardiomyopathy, in contradistinction to the effectiveness of A-ECG scoring in this study. Finally, although there was a modest, statistically significant correlation between the results (and changes in) the A-ECG score for LVSD in this study and those in echocardiographic LV ejection fraction, we agree with the general notion that clinically, one should not attempt to use ECG measures as linear surrogates for purely anatomical phenomena like echocardiographically estimated LV ejection fraction (as in our study) or mass (as noted by Bacharova) [27].

ECG, by its very nature, measures cardiac electrical phenomena and thus estimates changes in cardiac electrical status, not mechanical status. And while clinically speaking, changes in cardiac electrical and mechanical function often accompany and correlate with one another, they are not the same thing, nor is it necessarily helpful to consider them the same thing. For example, a degree of “left ventricular electrical remodelling” that can be ascertained from a change in an electrical phenomenon (such as a change in the spatial QRS-T angle, one of the components of the 5-parameter A-ECG score for LVSD evaluated in this study) [17] may actually have more prognostic importance than one ascertained from a mechanical phenomenon, especially at relatively equivalent levels of mechanical heart failure [28]. In addition, A-ECG combined with additional clinical information, such as genomic, proteomic and metabolomic biomarkers, might lead to a new appreciation of heart failure and its intermediate pathophenotypes. Notably 18% of our control patients also had conditions wherein genetic information would have aided diagnosis.

Although ECGs are often repeated over serial time points, the ability to assess, in detail, changes in one person’s conventional ECG in a single visualisation generally requires not only computerisation and dedicated software, but also the serial use of a single manufacturer's ECG platform. In a previous study [20], we demonstrated that analysis of A-ECG results by linear discriminant analyses can also allow for visualisation of changes in one person’s A-ECG condition over time in a fashion that is independent of the ECG manufacturer or platform. For population studies, including the present one, the same visualization technique can also have utility in direct comparison of ECG results between individuals, the identification of diseases or patterns of potential concern in groups of individuals, and in identifying isolated conditions in single patients. In this study, although the control patients were identified as having normal echocardiograms, it was evident from the A-ECG discriminant analysis, and also often verified clinically, that a number of these patients nonetheless had significant cardiac pathology, some of which had not been detected during their visits through tertiary care centres. This appeared to be particularly apparent for suspected hereditary conditions, such as the Long QT syndrome, though this diagnosis is yet to be confirmed in the aforementioned patients.

In single patients A-ECG also demonstrated an ability to identify trajectories towards greater health or disease based on a comparison of A-ECG-related results with those from echocardiography. Previous studies have shown that standard ECG parameters alter, in the short term, with diuresis in patients with heart failure [29]. In the Framingham heart study, ECG evidence of LV hypertrophy was shown to not only correlate with blood pressure but also changed with time [30]. The ECG changes were also predictive of subsequent cardiovascular events. The ability of the A-ECG to more objectively track changes than strictly conventional ECG may have utility in management decisions, such as helping to determine when further imaging or tailored treatment is appropriate. Given the cost-effectiveness and improved access of ECG compared with cardiac imaging, this improved upstream accuracy may lead to improved downstream decision making, reducing the costs of unnecessary investigations. In addition the distributed application of digital A-ECG lends itself well to telemedicine initiatives with greater community and primary care presence. In relation to this, whilst the A-ECG score that was further validated in this study clearly requires the derivation of several sophisticated ECG parameters, this does not necessarily imply that for its clinical application, the logic and signal processing behind the score must be directly programmed into the computer software or firmware of the various ECG machine manufacturers. For example it is already possible for any clinician to obtain such score results for his patients today, currently within hours, but in the future presumably within minutes, simply by uploading the patient’s raw digital ECG data file(s) to a secure central server site (see e.g., https://aecg.ch).

## 5. Conclusions

We have demonstrated that A-ECG scoring, a novel method of analysis of standard 12L ECG, has high sensitivity and specificity for detecting mechanical LVSD in a cohort with NICM, exceeding that described in previous studies. The A-ECG method also identified directional changes in mechanical LVSD over time which agreed with serial echocardiography findings in 76% of cases. In addition visualisations of multivariate A-ECG results by means of discriminant analyses were useful for identifying patients in the control group with other cardiac disease, notably those with possible hereditary conditions consistent with patient symptomatology. The high sensitivity and specificity of A-ECG, as well as its breadth of diagnostic ability, might make it an ideal population screening tool and method for triaging patients for noninvasive imaging. This may particularly be the case when used in conjunction with disruptive low cost imaging methods, such as pocket echocardiography [31]. Fully prospective application of this technology should be trialled in the clinical setting with human supervision to assess its utility and cost-effectiveness as both a diagnostic and screening tool.

### Limitations

The number of patients in this study was limited and the patient and control groups were not well matched for age, for the presence of atrial fibrillation and LBBB, and for medication use (drugs like digitals and amiodarone certainly also affecting the ECG). As ECGs were analysed retrospectively as part of clinical care they often occurred at time intervals distant to the echocardiogram. It is possible therefore that there would have been a change in ejection fraction over that time, partially accounting for the variability seen in Figure 1. Patients with ischemic cardiomyopathy were excluded and therefore the results may not be applicable to these patients. The reason for excluding them however was to evaluate the effectiveness of A-ECG in population of NICM patients without surrogate indicators of probable LVSD, such as widespread Q waves, *etc*. Although no scar was visible on most MRIs, only 26 patients underwent a coronary angiogram, such that potential contributions to LVSD by ischemia could not be fully excluded in all patients. Due to workload pressures and funding constraints, some ECGs were also not assessed by the human readers. However in relation to this, it should be noted that A-ECG is not currently applicable as a screening method for patients with IVCD or LBBB, an echocardiogram usually being immediately indicated for such patients based on their conventional ECG findings alone. A-ECG is also not intended to be a substitute for echocardiography, which provides a wealth of additional information such as chamber size, valvular function, *etc*, but is rather more suitable as an augmentation to traditional methods for sequential diagnostic testing, in the assessment of undifferentiated patients [31].
